# HMT-Net: A Multi-Task Learning Based Framework for Enhanced Convolutional Code Recognition

**DOI:** 10.3390/s26020364

**Published:** 2026-01-06

**Authors:** Lu Xu, Xu Chen, Yixin Ma, Rui Shi, Ruiwu Jia, Lingbo Zhang, Yijia Zhang

**Affiliations:** 1 School of Information Science and Technology, Zhejiang Sci-Tech University, Hangzhou 310018, China; xulu@zstu.edu.cn (L.X.); 2023220704010@mails.zstu.edu.cn (X.C.); 202220702019@mails.zstu.edu.cn (Y.M.); 2023220704058@mails.zstu.edu.cn (R.S.); 2 Donghai Laboratory, Zhoushan 316000, China; jiaruiwu@donghailab.com

**Keywords:** deep learning, multi-task network, convolutional code parameter recognition, channel coding identification

## Abstract

Due to the critical role of channel coding, convolutional code recognition has attracted growing interest, particularly in non-cooperative communication scenarios such as spectrum surveillance. Deep learning-based approaches have emerged as promising techniques, offering improved classification performance. However, most existing works focus on single-parameter recognition and ignore the inherent correlations between code parameters. To address this, we propose a novel framework named Hybrid Multi-Task Network (HMT-Net), which adopts multi-task learning to simultaneously identify both the code rate and constraint length of convolutional codes. HMT-Net combines dilated convolutions with attention mechanisms and integrates a Transformer backbone to extract robust multi-scale sequence features. It also leverages a Channel-Wise Transformer to capture both local and global information efficiently. Meanwhile, we enhance the dataset by incorporating a comprehensive sequence dataset and further improve the recognition performance by extracting the statistical features of the sequences. Experimental results demonstrate that HMT-Net outperforms single-task models by an average recognition accuracy of 2.89%. Furthermore, HMT-Net exhibits even more remarkable performance, achieving enhancements of 4.57% in code rate recognition and 4.31% in constraint length recognition compared to other notable multi-tasking frameworks such as MAR-Net. These findings underscore the potential of HMT-Net as a robust solution for intelligent signal analysis, offering significant practical value for efficient spectrum management in next-generation communication systems.

## 1. Introduction

The recognition of convolutional code parameters is crucial in scenarios such as non-cooperative communication and adaptive coding. Rice [1] was the first to propose a method for identifying 1/n convolutional codes through an algebraic approach that exploits the algebraic properties of the encoder and its dual code to ascertain encoder parameters and the generator matrix. Building upon this foundation, Filiol [2] extended the methodology to facilitate parameter recognition over finite fields GF(q). However, both approaches exhibit high sensitivity to noise and entail significant computational complexity. To mitigate these challenges, Liu [3] proposed a blind recognition algorithm based on the Walsh–Hadamard Transform (WHT), which established a more robust decision threshold and reduced computational complexity while enhancing performance in high bit error rate environments. Chen [4] further combined WHT with parity-check equation decomposition to identify punctured convolutional encoders with a code rate of (n−1)/n in noisy environments. Although this approach extended the application scope of WHT, it necessitates considerable computational resources, which may affect real-time performance.

With the advent of deep learning, the field of channel processing has embraced new opportunities for intelligent automation. Qin [5] introduced a TextCNN-based approach for channel code identification, treating received sequences as sentences to automatically extract features. This eliminated the need for manual feature engineering but showed restricted generalization across different code types. Wang [6] proposed a Deep Residual Network (DRN) using soft-decision sequences to enhance adaptability, yet this method assumes the sequence originates from the start of a codeword, limiting its robustness against synchronization errors or interference. To handle more complex environments, Tian [7] and Dehdashtian [8] developed deep learning methods for blind recognition under fading conditions, while Yang [9] incorporated multi-scale CNNs to specifically capture features from various puncturing matrices.

In recent years, the integration of multi-task learning (MTL) with signal processing has opened up new avenues for joint parameter recognition. Research by Wang [10] and Jagannath [11] demonstrated that MTL significantly improves robustness in modulation classification and 5G deployment by extracting inter-task correlations. Liu [12] proposed the BERD receiver, enabling joint processing of channel estimation, modulation recognition, and code identification. Similarly, MTL frameworks have been explored in RF fingerprinting [13], semantic communication [14], and space–time block code recognition [15]. However, many existing MTL-based methods, such as those employing BiLSTM-CNN architectures [16], remain limited to a narrow range of code types or exhibit performance degradation at low signal-to-noise ratios (SNR).

More recently, researchers have explored advanced architectures to capture long-range dependencies. Dao [17] introduced a Vision Transformer-based model for modulation classification, and Olaniyi [18] utilized attention-based Turbo-Autoencoders for improved reconstruction. Furthermore, Mao [19] explored foundation models for blind recognition. While attention mechanisms like Channel-Wise Transformer [20] and Dilated Channel Attention [21] have achieved success in domains like object detection and dead reckoning, their potential in joint convolutional code parameter recognition remains largely underexplored.

Despite these advancements, a critical gap remains: most existing methods treat code rate and constraint length recognition as isolated tasks or rely on structures that struggle to balance local features with global dependencies. To address these challenges, we propose a multi-task learning-based framework named HMT-Net (Hybrid Multi-Task Network). Distinct from existing frameworks relying on standard convolutions or recurrent units, HMT-Net introduces a novel hybrid architecture combining Dilated Channel Attention (DCA) and Channel-Wise Transformer (CWT). The primary contributions of this work are summarized as follows:

1. Novel Hybrid Architecture Design: We propose a feature extraction module integrating DCA and CWT. This structure expands the receptive field to capture global features under low SNR conditions while utilizing channel-wise attention to isolate task-specific features for code rate and constraint length recognition.
2. Dataset Enhancement Strategy: We construct a comprehensive sequence dataset incorporating statistical features. This enhancement significantly improves the model’s discriminative power and robustness against channel noise.
3. Performance Advantages: HMT-Net can recognize a broader set of convolutional code parameters, covering high code rates and approximately constrained lengths. Experimental results demonstrate that this model outperforms multiple state-of-the-art signal recognition frameworks in terms of recognition accuracy.

The remainder of this paper is structured as follows: Section 2 provides the system model and principles of convolutional codes. Section 3 elaborates on the dataset generation and the HMT-Net architecture. Section 4 presents the performance evaluation. Finally, Section 5 concludes the paper and discusses future directions.

## 2. System Model

In this section, we present the overall framework of the proposed research. First, we introduce the digital communication system model, including the signal generation and channel environment, to establish the context for signal simulation. Subsequently, we formulate the multi-task recognition problem by defining the mathematical relationships between the input signals and the target parameters.

### 2.1. Digital Communication System

**Figure 1:**
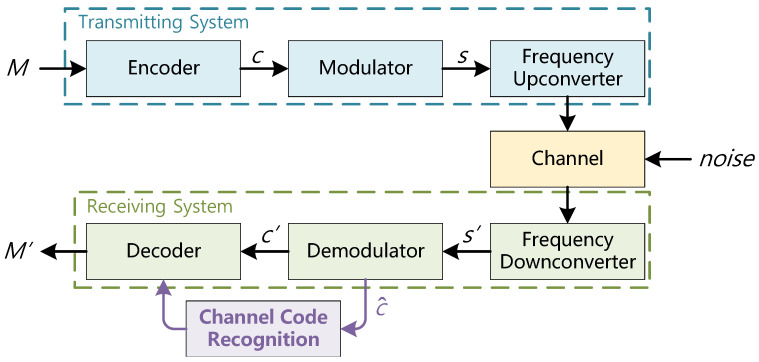
Digital communication system block diagram.

In this study, we propose a signal transmission system, as shown in Figure 1. specifically designed for the identification of channel coding parameters under low SNR conditions.

We adopt convolutional coding [22] as the channel coding method, denoted by C(n,k,l), where n represents the number of output bits per input, *k* is the number of input bits, and *l* is the constraint length, indicating the depth of memory used in encoding past input bits. Convolutional codes can be represented using a generator matrix.

After channel coding, Binary Phase Shift Keying (BPSK) is chosen as the modulation scheme. The transmitted signal *s* passes through the channel, which is assumed to be an additive white Gaussian noise (AWGN) channel. During demodulation, soft-decision decoding is applied to the received signal. It is worth noting that the proposed recognition framework operates on demodulated soft-decision sequences, and is therefore not inherently tied to a specific modulation scheme or channel model.

The obtained soft information $\hat{c}$ is then analyzed using recognition algorithms to identify the convolutional coding parameters.

### 2.2. Multi-Task Recognition System

The multi-task recognition system is specifically designed to analyze convolutional codes. A multi-task dataset can be defined as a triplet, as shown by Equation (1).

(1) $$D=\left(R,Y,T\right)$$

where R⊆R is the sample space, each sample belongs to a task set $T=\{\tau_{1},$…$,\tau_{m}\}$, representing distinct tasks. For each task $\tau_{k}$, there exists a corresponding label space $Y={⊗}_{k=1}^{m}C_{k}$, where ⊗ denotes the Cartesian product and $C_{k}$ is the label set for task $\tau_{k}$.

This study adopts data-driven deep feature extraction and leverages neural networks to directly learn classification boundaries. Additionally, an adaptive loss weighting mechanism is introduced to adjust the impact of each task based on its respective loss, thereby addressing class imbalance and improving prediction accuracy across tasks. The final prediction is given by Equation (2).

(2) $$\hat{y}=\arg\max_{y\in Y}f_{\mathrm{HMT}}\left(y|r;\theta \right)$$

where, $f_{\mathrm{HMT}}\left(y|r;\theta \right)$ is the model based on HMT-Net, and it outputs the predicted labels by optimizing parameters θ to maximize classification accuracy.

## 3. Proposed Approach

In this section, we provide a detailed description of the proposed HMT-Net framework. We begin by introducing the overall network architecture, outlining its four hierarchical components. Next, we detail the data processing strategy, followed by an in-depth discussion of the shared feature extraction module and the task-specific modules. Finally, we introduce the dynamic weight adjustment mechanism designed to optimize the multi-task training process.

### 3.1. HMT-Net

**Figure 2:**
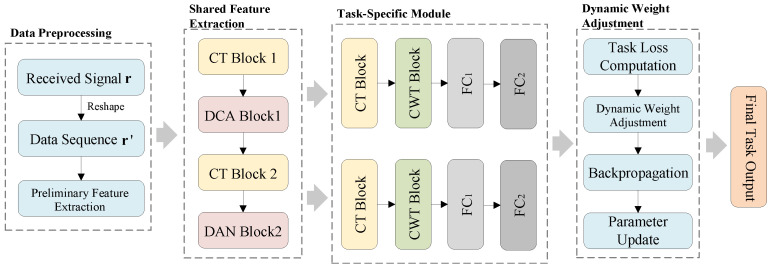
HMT-Net Model.

The HMT-Net architecture consists of four core components, as shown in Figure 2. Firstly, the data preprocessing stage completes the feature pre-extraction. Secondly, in the shared feature extraction module, DCA (Dilated Channel Attention) and CT (Convolutional Transformer) modules are introduced to hierarchically capture spatial features across different dimensions of the input sequence, as well as the interdependencies between channels. Thirdly, in the task-specific modules, a hybrid structure combining Channel-Wise Transformer (CWT) and CT modules is adopted to further improve the network’s capability in parsing various convolutional code parameters. Finally, the dynamic loss weighting module performs real-time monitoring to balance computational resources across tasks.

#### 3.1.1. Data Preprocessing Module

**Figure 3:**
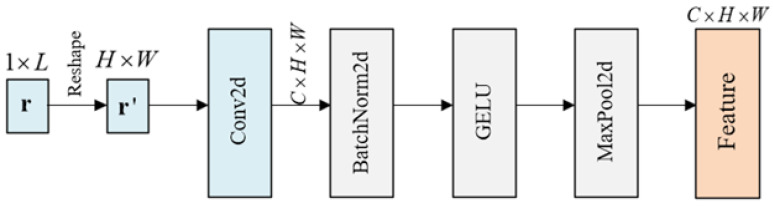
Structure of the data preprocessing module.

As shown in Figure 3, the data preprocessing module transforms the original soft-decision samples of convolutional codes from a one-dimensional format into a two-dimensional representation through matrix reshaping. This operation spatially compacts the features, thereby enhancing their suitability for neural network-based feature extraction.

The feature preprocessing module consists of 2D convolutional layers, batch normalization, Gaussian Error Linear Unit (GELU) activation, and max pooling. Among them, the 2D convolutional layer serves as the core structure for local feature extraction and channel expansion, providing a foundation for subsequent classification tasks. After convolution, the extracted feature tensor of shape 1×H×W is transformed into C×H×W, where *C* is the number of output channels. Batch normalization(BN) helps accelerate training, mitigate gradient vanishing/explosion, and enhance generalization. Max pooling is used to reduce computational cost by downsampling the features.

#### 3.1.2. Shared Feature Module

The shared feature module consists of DCA and CT modules.

**Figure 4:**
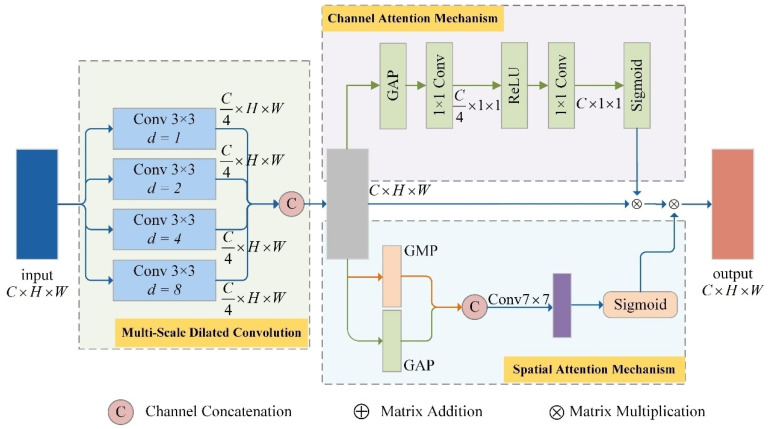
DCA Module.

The internal structure of the DCA module is illustrated in Figure 4. The mechanism originally proposed in [23], is a feature enhancement approach that integrates multi-scale dilated convolutions with both channel attention and spatial attention mechanisms. It enables the extraction of multi-scale features from convolutional sequences, thereby enhancing the capability to capture information across various receptive fields, and improving the fusion of local and global features—ultimately boosting performance in detection tasks. In this study, we have improved the DCA module to accommodate the recognition of convolutional codes with higher code rates and larger constraint lengths by expanding the coverage range of its original multi-scale dilated convolutions.

In the DCA structure diagram, GAP (Global Average Pooling) is used to calculate the average feature value across the channel dimension, as expressed in Equation (3).

(3) $$F_{GAP}=\frac{1}{H\times W}\sum_{i=1}^{H}\sum_{j=1}^{W}F_{i,j}$$

Meanwhile, GMP (Global Max Pooling) is utilized to determine the maximum value along the channel dimension in order to extract the most strongly activated feature from each channel, as shown in Equation (4).

(4) $$F_{GMP}=\max_{i\subset \left[1,H\right],j\subset \left[1,W\right]}F_{i,j}$$

where *i* and *j* represent the positional indices of height and width on the feature map, respectively.

**Figure 5:**
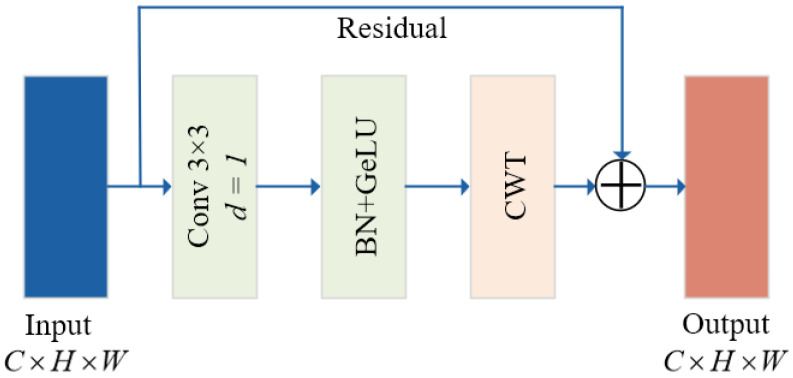
CT module structure schematic diagram.

The structure of the CT module is schematically shown Figure 5. It consists of two components: the Convolutional Layer and the Transformer [24], which work together to enhance feature extraction while employing residual connections to improve training stability. Notably, the Transformer module primarily utilizes the CWT to manage global attention within the channel dimension. This aspect will be elaborated upon and dissected in detail in the subsequent subsection, as CWT serves as a fundamental element of the task-specific module.

#### 3.1.3. Task-Specific Modules

The task-specific modules mainly include CT module, CWT module, fully connected (FC) modules, denoted as FC1 and FC2. Among these, the FC1 module elevates the *C*-dimensional features to r×C-dimensions, thereby augmenting the model’s expressive capacity. Subsequently, it connects the GELU activation function to introduce nonlinearities, which endows the model with a more robust feature transformation capability. The FC2 module further maps these feature dimensions to correspond with the number of categories in the final individual task, facilitating classification decision for each subtask.

**Figure 6:**
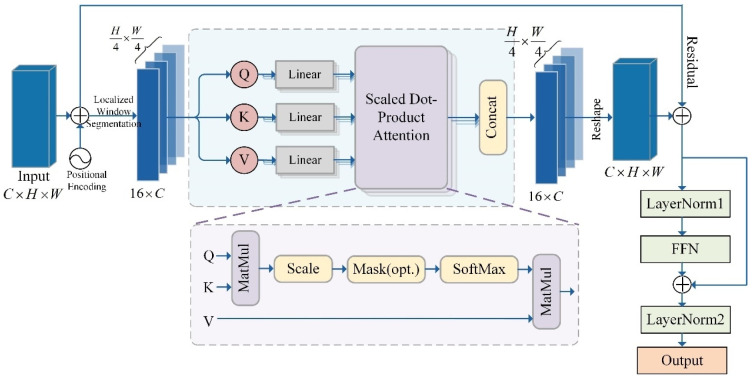
CWT module structure schematic diagram.

The structure of the CWT module is shown in the schematic diagram of Figure 6. The CWT module is a variant of an efficient attention mechanism that achieves efficient characterization of image features by integrating local window attention with channel-level feature interaction. Compared to the standard Transformer, the CWT employs a window partitioning strategy to mitigate computational complexity and focuses on local region dependencies through scaled dot product attention.

For the input feature map $X\in R^{C\times H\times W}$, the CWT firstly encodes its spatial location and conveys this information through a learnable parameter $P\in R^{C\times H\times W}$. This process enhances the attention computation by making it spatially aware. Then, a local windowing technique with a stride of 4 is employed on the features, thereby segmenting the feature map into smaller windows of $\left(H/4\right)\times \left(W/4\right)$. For each window $X_{i}$, the CWT uses a multi-head self-attention mechanism for in-channel feature modeling. The input features are projected to query (Q), key (K), and value (V) representations. The scaled dot product attention is calculated as shown in Equation (5).

(5) $$A=soft\max\left(\frac{QK^{T}}{\sqrt{d_{k}}}\right)$$

where, $d_{k}=C^{\prime }$ is a scaling factor to stabilize the gradient and prevent extreme values from affecting the calculation. Attention weighted summation yields the output, as defined in Equation (6).

(6) Z=AV

In normalized and feed-forward networks (FFN), the module takes the computed attention result Z through residual concatenation and normalization. The CWT then employs the FFN for channel-level feature enhancement. In particular, the FFN consists of two 1 × 1 convolutional layers and the GELU activation function.

The advantage of CWT through visual feature modeling lies in its utilization of local perceptual capabilities via window partitioning. This approach allows the attention computation to concentrate on local region, thereby enhancing feature capture capability, while simultaneously reducing the computational overhead with global attention. Consequently, it is particularly well-suited. Moreover, the CWT places significant emphasis on channel information during attention computation, facilitating a more comprehensive establishment of dependencies among different feature channels. This channel-level interactions serve as a bridging within the CWT, enabling the fusion of local perceptual capabilities with broader global information. This characteristic aligns with the properties of convolutional codes, which can extract the short-term features and integrate them to derive long-term feature. As a result, local features benefit from global information, while global insights enhance local perceptual abilities through channel interactions. Furthermore, this framework achieves an equilibrium between performance and computational complexity.

#### 3.1.4. Dynamic Weight Adjustment

In multi-task learning, the network needs to optimize the objective function for multiple tasks simultaneously. However, the losses associated with different tasks may exhibit variations in scale and differing convergence rates. This situation is particularly prevalent in the multi-task learning of convolutional codes, as the recognition difficulty related to constraint length and code rate varies across different situations. Consequently, the dynamic weight adjustment strategy emerges as an effective intelligent tuning strategy that can effectively mitigate these challenges, thereby enhancing both the efficiency and stability of multi-task training.

For *N* tasks, we define the loss associated with each task as $L=[L_{1},L_{2},\ldots ,L_{N}]$, where $L_{i}$ represents the loss of the *i* task. The objective of dynamic loss weighting in multi-task learning frameworks is to enhance the optimization process across all tasks by adaptively adjusting task weights based on the percentage of changes in loss. The initial baseline loss for each task is defined as shown in Equation (7).

(7) $$L_{i}^{\left(0\right)}={\mathrm{init\_losses}}_{i}$$

where $L_{i}^{\left(0\right)}$ represents the initial baseline loss for the *i* task, and ${\mathrm{init\_losses}}_{i}$ denotes the pre-recorded initial loss value obtained from the first training iteration.

Subsequently, the ratio of each task loss relative to the baseline loss will be calculated as shown in Equation (8).

(8) $$r_{i}=\frac{L_{i}}{L_{i}^{\left(0\right)}}$$

where $r_{i}$ denotes the relative loss ratio for task *i*, and $L_{i}$ represents the real-time loss value computed in the current training step.

In order to prevent extreme cases from the weight update, upper and lower cutoffs (clamping) were used, as shown in Equation (9).

(9) $$r_{i}^{\prime }=\mathrm{clamp}\left(r_{i},0.1,10.0\right)$$

where $r_{i}^{\prime }$ is the clamped ratio, and the function clamp(·) restricts the value of $r_{i}$ to the range [0.1,10.0] to avoid numerical instability caused by outliers.

The normalized $r_{i}^{\prime }$ keeps the sum of relative contributions of multiple tasks stable, as described in Equation (10).

(10) $$w_{i}^{\prime }=\frac{r_{i}^{\prime }}{\sum_{j=1}^{N}r_{j}^{\prime }+10^{-6}}$$

where $w_{i}^{\prime }$ represents the normalized weight for the *i* task, *N* is the total number of tasks, and $10^{-6}$ is a small constant (ϵ) added to the denominator to prevent division by zero.

To enhance the smoothness of weight adjustments, this study incorporates the EMA (Exponential Moving Average) [25]. This approach allows for a combination of historical weights $w_{i}^{old}$ and newly computed weights $w_{i}^{\prime }$ to generate an updated set of weights $w_{i}^{new}$, calculated as shown in Equation (11).

(11) $$w_{i}^{new}=0.8\cdot w_{i}^{old}+0.2\cdot w_{i}^{\prime }$$

where $w_{i}^{new}$ denotes the updated weight used for the next iteration, and $w_{i}^{old}$ is the weight from the previous step. The coefficients 0.8 and 0.2 represent the momentum factor and the update rate, respectively, controlling the speed of weight adaptation.

Weighted losses can be calculated using the new dynamic weights, as shown in Equation (12).

(12) $$L_{adaptive}=\sum_{i=1}^{N}w_{i}^{new}\cdot L_{i}$$

where $L_{adaptive}$ is the final weighted total loss used for backpropagation, summing the product of the dynamic weight $w_{i}^{new}$ and the task loss $L_{i}$ across all *N* tasks.

This EMA dynamic loss weighting module assigns greater weight to the most recent data, while the influence of historical data decays exponentially. Consequently, historical data will have a diminishing impact on the optimization process over time. This approach smooths gradient changes and enhances convergence speed, thereby increasing the stability of the optimization process. It is particularly suitable for long-term trend modeling applications, such as convolutional codes in time series analysis, and it improves the model’s generalization capability.

It is worth noting that while other adaptive weighting methods exist, such as GradNorm and Uncertainty-based weighting, the proposed EMA strategy offers distinct advantages in this specific context. GradNorm typically requires computing gradients for auxiliary weights at each iteration, introducing additional computational overhead. Similarly, Uncertainty-based weighting relies on optimizing learnable parameters that can be sensitive to data volatility. In contrast, the EMA-based approach provides a lightweight, parameter-free solution by smoothing loss trends. This mitigates the impact of transient loss fluctuations while enabling stable multi-task optimization without increasing training overhead.

#### 3.1.5. HMT-Net Parameterization

The preceding sections describes the overall network architecture of HMT-Net, and Table 1 provides a detailed illustration of its specific structure along with the parameter configurations of each module.

Table 2 presents the detailed hyperparameter configurations used during network training. In addition, the study implements an early stopping strategy to terminate training when the validation loss shows no improvement over 10 consecutive epochs. The specific parameters of the ReduceLROnPlateau learning rate scheduler are as follows: the learning rate adjustment is triggered when the validation loss reaches a plateau, and it is reduced by a factor of 0.2. If the validation loss does not decrease significantly within a single training cycle, a learning rate decay is applied.

### 3.2. Data Processing

This study focuses on identifying the code rate and constraint length of the convolutional code. The type of convolutional code used is the front feedforward unsystematic convolutional code, which exhibits robust error correction performance and strong anti-jamming ability. The detailed configuration of the parameters for the convolutional code utilized in this experiment is listed in Table 3.

The classes of convolutional codes examined in this study encompass a range of typical configurations with varying complexity. These configurations span from simple coding forms characterized by low code rates and short constraint lengths to more intricate coding structures featuring high code rates and long constraint lengths. The objective is to thoroughly assess the adaptability and generalization performance of the proposed model in multi-class convolutional code recognition tasks.

In the specific construction of the dataset, the study establish the signal-to-noise ratio (SNR) range from −20 dB to 20 dB, sampling at intervals in 2 dB. For each set of convolutional coding parameters, 1000 sets of samples are generated for each SNR point; among these, 80% are allocated for model training and the remaining 20% for model validation. The entire dataset comprises 504,000 training samples and 126,000 test samples. Each sample has a length of 256 and is dimensionally transformed to format of 1×16×16 to facilitate processing by the network. This dataset is meticulously designed to ensure the model’s ability to generalize under different SNR conditions, while providing sufficient data support for the convolutional code recognition task to improve robustness and accuracy.

**Figure 7:**
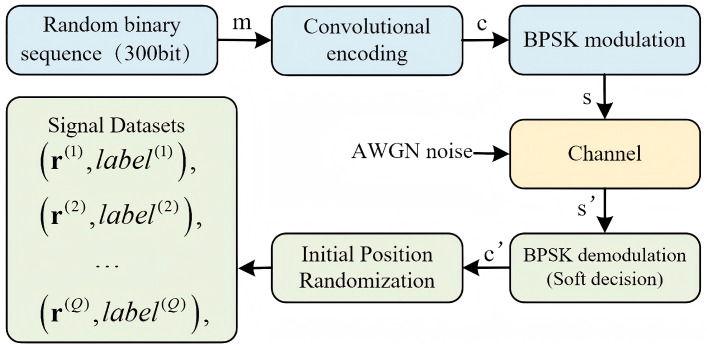
Soft judgment dataset generation.

The procedure for generating samples of the convolutional code time series dataset is illustrated in Figure 7 as follows:

1. Generate a binary random sequence *m*, with length of 300 bit.
2. The corresponding convolutional encoding is performed according to the parameters specified in Table 1.
3. BPSK modulation is used to modulate the coded sequence to generate the modulated signal *s*.
4. Addition of AWGN produces a noisy signal and simulates the effect of channel transmission $s^{\prime }$.
5. The channel is demodulated to obtain a soft verdict affected by interference $c^{\prime }$.
6. From the first 0 to 20 digits of the sequence, one of them is randomly selected as the starting point, and 256 bits are extracted as the sample sequence *r*, and the label is added to it at the same time.
7. Repeat steps 1–6 to generate the complete dataset, as formulated in Equation (13).

(13) $$\left(\Omega_{r},\Omega_{label}\right)=\left\{\left(r^{\left(1\right)},label^{\left(1\right)}\right),\ldots ,\left(r^{\left(Q\right)},label^{\left(Q\right)}\right)\right\}$$

where $\Omega_{r}$ and $\Omega_{label}$ denote the set of coded signal samples and the corresponding set of labels, respectively, with an ensemble size of *Q*, and $\left(r^{\left(q\right)},label^{\left(q\right)}\right)\left(1⩽q⩽Q\right)$ denotes the *q*-th labeled sample in the training set.

To further enhance the feature extraction of the sequences, the dataset incorporates statistical feature extraction techniques based on autocorrelation analysis, WHT and its variants. This approach enables the model to more accurately capture both the regularity and discriminative features of the coded sequences, which we refer to as the comprehensive sequence dataset. The dataset consists of a series of feature extraction operations performed on the basis of 256 bits of convolutionally encoded signals that are randomized at the initial location of the interception.

Enhanced autocorrelation features include both base autocorrelation and differential autocorrelation. Base autocorrelation is sensitive to the periodicity of the signal and pattern similarity, making it particularly suitable for analyzing the features of convolutional codes. In contrast, differential autocorrelation captures the autocorrelation trend of the signal, thus providing additional discriminative power. The study will obtain nine base autocorrelation feature value, in addition to eight differential autocorrelation values. Together, these elements comprise a total of 17 enhanced autocorrelation features.

The WHT is a discrete signal transform that resembles to the Fourier transform; however, it relies solely on addition and subtraction. This characteristic allows for rapid computation, making the WHT particularly advantageous for processing binary data. Nevertheless, this study aims to leverage soft verdict signals for parameter identification of convolutional codes. Consequently, the WHT spectrum may not exhibit as intuitive as binary inputs do, and requires analysis alongside statistical features such as mean, standard deviation, maximum, minimum, 25% and 75% quantiles, L1 and L2 norms, and high-frequency energy ratio. This includes 9 features of WHT spectral statistics.

**Figure 8:**
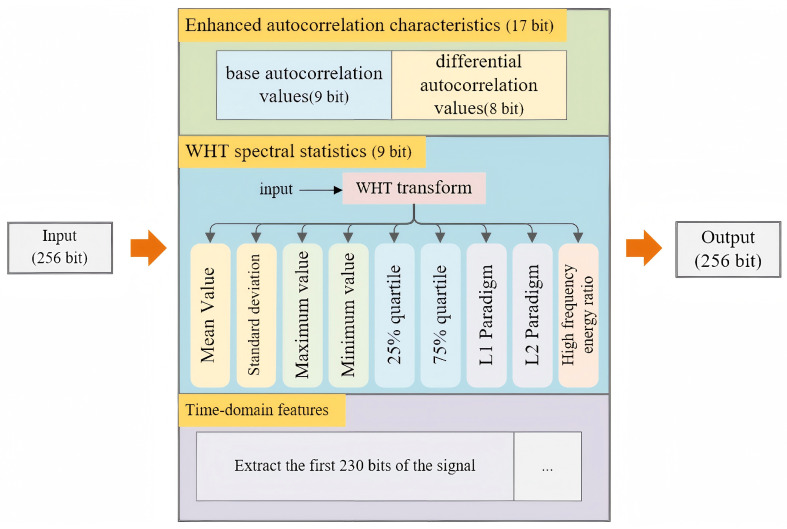
Generation of the comprehensive sequence dataset.

In summary, through the application of mathematical statistics, the study obtains 26 features that will serve as the initial 26-bit sequence of the dataset. These features will be combined with additional 230 bits of intercepted information sequences to form a 256 bits feature extraction dataset. Figure 8 illustrates the flowchart for the generation of synthesized sequences of the dataset.

## 4. Experimental Results

In this section, we present a comprehensive evaluation of the proposed HMT-Net framework. We begin by analyzing the effectiveness of the multi-task learning strategy through comparative experiments against single-task and combined-task baselines. Subsequently, we benchmark HMT-Net against state-of-the-art multi-tasking frameworks to demonstrate its superior performance. We further investigate the impact of the dynamic loss weighting module and different dataset configurations on recognition accuracy. Finally, ablation studies are conducted to validate the contribution of each core component within the network architecture.

### 4.1. Multi-Task Effectiveness Analysis

To evaluate the effectiveness of HMT-Net in multi-task recognition of convolutional code parameters, this section presents a series of comparative experiments conducted across three recognition modes, namely, single-task, combined-task, and multi-task. These experiments are designed to assess model performance in terms of recognition accuracy, confusion matrix, and other relevant metrics, thereby validating the superiority of the proposed method. To ensure consistency and fairness in experimental conditions, the single-task learning model is derived from the HMT-Net architecture by retaining only its feature-sharing module and single-task branch; this modified architecture is referred to as HST-Net (Hybrid Single Task Network). In the combined task recognition scheme, the independent classification features from two separate HST-Net are concatenated and subsequently fed into the classifier for joint recognition, forming the HCT-Net (Hybrid Concatenation Task Network) structure. All experiments are based on the same dataset construction strategy and hyper-parameter settings, ensuring a fair comparison of the performance differences among the network architectures in the joint recognition task.

**Figure 9:**
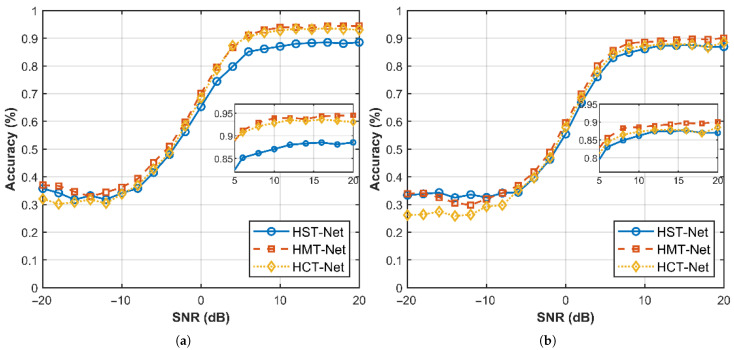
Comparison of single-task recognition results of HST-Net, HCT-Net, and HMT-Net. (**a**) Code rate recognition accuracy (**b**) Constraint length recognition accuracy.

Figure 9a illustrates the accuracy variation curves of HST-Net, HCT-Net and HMT-Net in recognizing the code rate of convolutional codes under different SNR conditions. HMT-Net consistently outperforms the other models across the entire SNR range. Specifically, HMT-Net achieves approximately 5% higher accuracy than HST-Net and 1% higher than HCT-Net under high SNR conditions. Moreover, HMT-Net maintains the best performance even in extreme SNR scenarios, with a relatively flat accuracy degradation curve as SNR decreases. As shown in Table 4, on the full-domain SNR, the code rate recognition accuracy of HMT-Net is 4.30% higher than that of HST-Net and 1.89% higher than that of HCT-Net, which clearly demonstrates the superiority of HMT-Net in this recognition task.

Figure 9b shows the accuracy variation curves of HST-Net, HCT-Net and HMT-Net in recognizing the convolutional code constraint length of convolutional codes under different SNR conditions. It is evident that HMT-Net nearly dominates across the entire SNR range, with only a minor performance lag behind HST-Net at SNR < −8 dB. As supported by the data in Table 4, HMT-Net achieves approximately 3% higher accuracy than HST-Net and over 1.5% higher than HCT-Net in the high SNR range. In the extreme SNR interval, the accuracy of HMT-Net is significantly higher than that of HCT-Net. As shown in Table 4, across the full domain SNR, the constraint length recognition accuracy of HMT-Net is 1.47% higher than that of HST-Net and 2.85% higher than that of HCT-Net, which clearly confirms its superiority in recognizing the constraint length of convolutional codes. For the average recognition accuracy, HMT-Net is 2.89% higher than HST-Net and 2.37% higher than HCT-Net.

**Figure 10:**
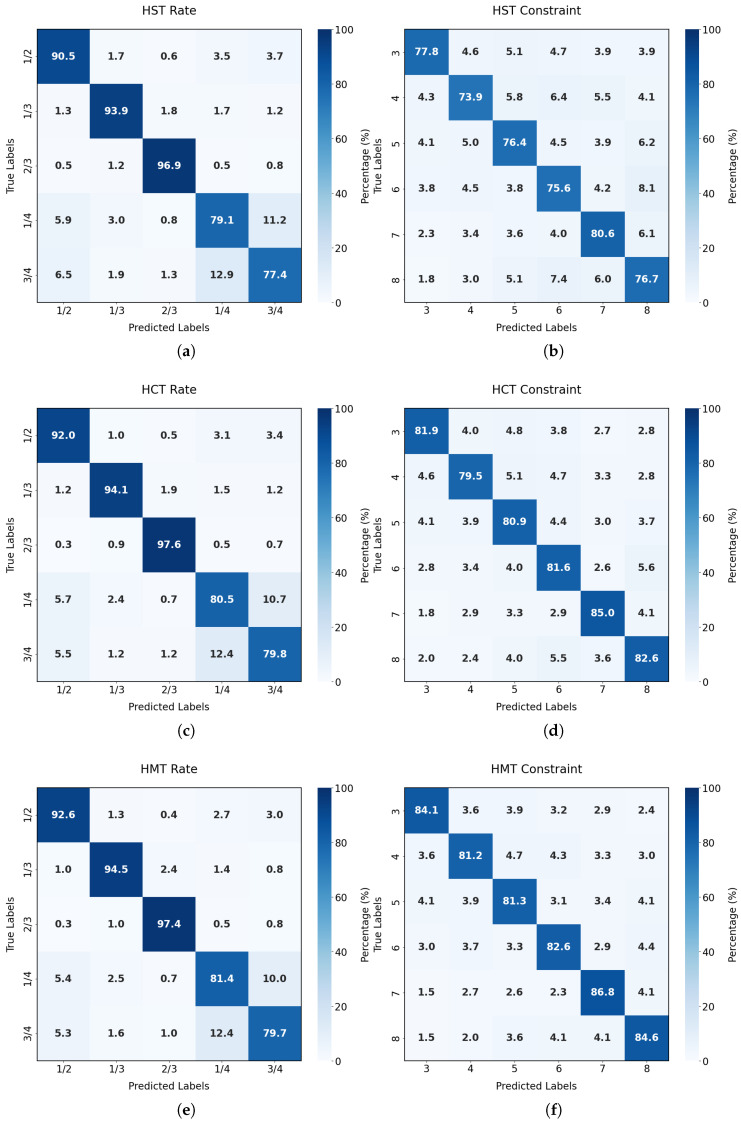
Confusion Matrix for HMT-Net, HCT-Net and HST-Net (**a**) HST-Net code rate confusion matrix (**b**) HST-Net constraint length confusion matrix (**c**) HCT-Net code rate confusion matrix (**d**) HCT-Net constraint length confusion matrix (**e**) HMT-Net code rate confusion matrix (**f**) HMT-Net constraint length confusion matrix.

In order to further analyze the recognition performance of HMT-Net, this study examines the confusion matrices of HMT-Net, HCT-Net, and HST-Net for the tasks of recognizing convolutional code rates and constraint lengths. These matrices are generated in the range of SNR ≥ 0 dB, as illustrated in Figure 10.

To further quantitatively evaluate classification performance, we analyzed derivative metrics of the confusion matrix—precision, recall, and F1 score—to compare the performance of HST-Net, HCT-Net, and HMT-Net. As shown in Table 5, HMT-Net consistently outperformed baseline models in macro-average F1 score. Specifically, in the overlapping feature space regions where HST-Net and HCT-Net underperformed, HMT-Net effectively reduced classification errors.

In conclusion, the confusion matrix further demonstrates the enhancement in accuracy achieved by HMT-Net through its implementation of the multi-task learning mechanism for convolutional code recognition. As illustrated by the confusion matrix, HMT-Net effectively reduces instances of misclassification across all scenarios while attaining an optimal level of accuracy consistently.

Table 6 presents a comparison of the number of parameters for HST-Net, HCT-Net, and HMT-Net on an identical hardware and software platform. The data indicate that HMT-Net significantly reduces the overall model size through a parameter-sharing mechanism while simultaneously executing joint recognition tasks. Its total number of parameters is considerably smaller than those in both single-task models and lower than that in the combined-task model, HCT-Net. Consequently, HMT-Net demonstrates enhanced efficiency during training and inference while preserving recognition accuracy. This model exhibits effective resource utilization, highlighting its strong potential for engineering applications in managing model complexity and validating structural design efficiency. The computational complexity analysis was performed on a server equipped with an AMD Ryzen 9 5950X CPU (AMD, Santa Clara, CA, USA) and an NVIDIA GeForce RTX 3090 Ti GPU (Nvidia, Santa Clara, CA, USA).The inference time and FLOPS were measured with a batch size of 128, and the timings account for the entire processing pipeline, including the data preprocessing module.

The results in Table 5 indicate that the proposed network effectively recognizes both parameters. However, varying degrees of confusion occur when the code rate is 1/4 or 3/4. This challenge likely arises from the higher randomness in the constituent sequences of long-code-length and high-code-rate convolutional codes, which makes feature extraction more difficult.

Against this backdrop, the EMA-based dynamic weight adjustment strategy plays a pivotal role in stabilizing multi-task learning. By smoothing loss fluctuations under low signal-to-noise ratio conditions, this strategy effectively mitigates the adverse effects of transient loss jitter, thereby promoting more robust and efficient parameter identification.

### 4.2. Performance Analysis of Multiple Networks

This subsection provides a comparative analysis of HMT-Net with established multi-tasking frameworks in the domain of signal recognition, specifically MAR-Net [26], AMSCN [27], FFMNet [28], and ConvLSTM-TFN [29]. This comparison aims to further elucidate the state-of-the-art capabilities of the HMT-Net model proposed in this chapter. Among these frameworks, MAR-Net employs a generalized joint identification approach for channel coding that leverages multi-scale null convolution, an attention mechanism, and a residual convolutional network. In contrast, AMSCN introduces a dual-task deep learning model founded on a shared attention mechanism to facilitate simultaneous modulation identification and specific transmitter source identification. Furthermore, FFMNet develops a self-encoder architecture designed for the compression and transmission of intermediate features within wireless collaborative intelligence scenarios utilizing multi-task learning in the channel. ConvLSTM-TFN, an innovative blind recognition network previously proposed by us, integrates convolutional layers, Long Short-Term Memory (LSTM) networks, and a self-attention mechanism.

**Figure 11:**
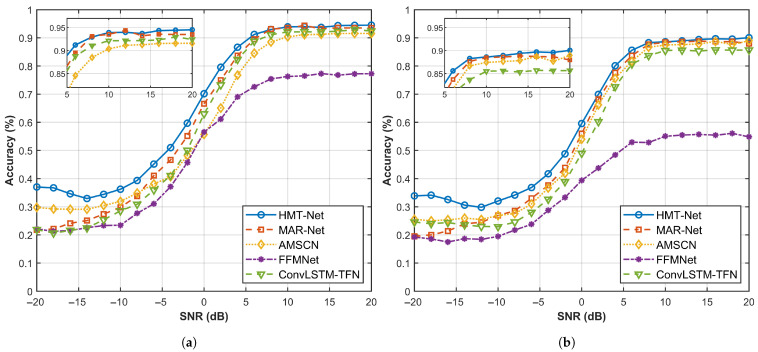
Convolutional code recognition accuracy comparison under multiple networks. (**a**) Rate Accuracy Comparison (**b**) Constraint Length Accuracy Comparison.

Figure 11 compares the experimental results of the parameter recognition accuracies across different multi-task learning frameworks. The findings indicate that the HMT-Net proposed in this study demonstrates superior performance in both high and low signal-to-noise ratio range. Among them, FFMNet excels at extracting macroscopic features; however, it struggles to adequately recognize types of constraint length, which are more temporal in nature [30]. The features associated with constraint length are tightly interwoven and complex, significantly impacting FFMNet’s feature extraction capabilities. Regarding code rate, the self-encoder may not effectively capture the periodicity and redundancy characteristics of the signal, which further compromises its recognition accuracy.

As shown in Table 7, among all the methods compared, HMT-Net exhibits the superior performance. At high SNR, it surpasses the second-best method, MAR-Net, by 1.85% and 1.84% in code rate recognition and constraint length recognition, respectively. Under global SNR, HMT-Net demonstrates even more remarkable performance, achieving improvements of 4.57% and 4.31% over MAR-Net in these same aspects.For the average recognition accuracy, HMT-Net is 4.44% higher than MAR-Net. This clearly substantiates the exceptional noise resistance capabilities and advanced structural design of the network presented in this study.

### 4.3. Effectiveness of Dynamic Loss Weighting Modules

The EMA dynamic loss weighting module employed in this study is designed to dynamically adjust the loss weighting ratio of each subtask through an exponential moving average. This adjustment is based on the rate of change of the loss function associated with each subtask, thereby facilitating more effective multi-task training.

In order to verify the effectiveness of this dynamic loss weighting module, Table 8 demonstrates a comparison of the recognition performance between the EMA method and the fixed weight setting method. Here, α and β refer to the fixed preset loss weights for the two sub-tasks in the fixed weight configuration, respectively. The experimental results indicate that incorporating the dynamic weighting mechanism of EMA leads to stable performance in convolutional code parameter recognition tasks, with recognition accuracy comparable to that achieved under optimal preset loss weight allocation α=0.4, β=0.6. Notably, the EMA method eliminates the need for manual adjustments to optimal weight assignments, thereby enhancing both training efficiency and stability.

### 4.4. Effect of Different Datasets on Recognition Performance

**Figure 12:**
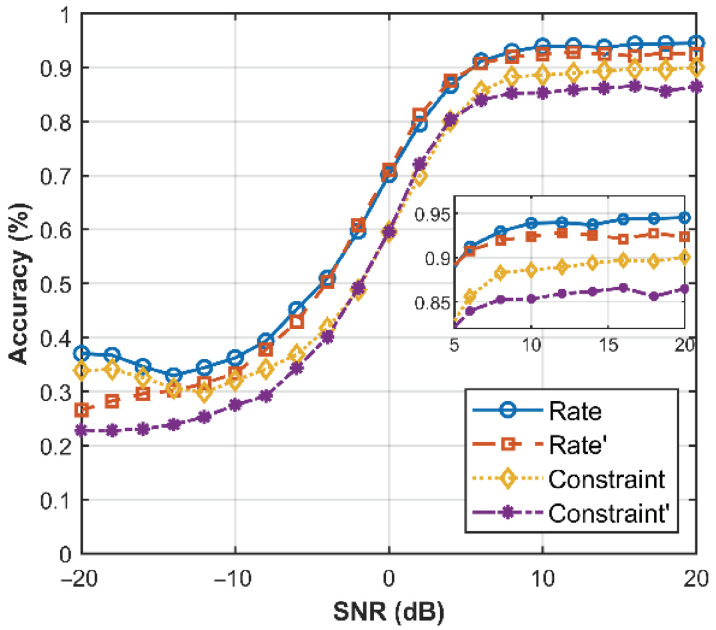
Effect of different datasets on the recognition performance. rate, Constraint is the recognition performance under the comprehensive sequence dataset; Rate′, Constraint′ is the recognition performance under the soft verdict dataset.

In order to explore the effect of feature extraction on the recognition performance of convolutional code parameters, we used two different datasets for comparison: one consisting the original 256-bit soft verdict sequence without any feature extraction applied, and the other comprising a 256-bit composite sequence that incorporates additional feature extraction information. The experimental results obtained from HMT-Net are shown in Figure 12.

It can be concluded that the comprehensive sequence dataset demonstrates a more significant improvement in identifying convolutional code parameters across most SNRs. Notably, the enhancement in identifying the constraint length is particularly identifying, characterized by an approximate 5% increase within the range of extremely poor SNRs. Furthermore, there is also a substantial improvement in the high SNR case. This underscores the superiority of the comprehensive sequence dataset.

### 4.5. Ablation Experiment

To investigate the effectiveness of each module within HMT-Net, this study conducts ablation experiments on DCA-Block, CT-Block, and CWT-Block by individually removing these components. This approach allows for a more thorough examination of the rationale behind the network structure. The results are shown in Table 9.

Baseline 1 denotes a network architecture that combines Backbone, CT-Block, and CWT-Block; Baseline 2 denotes a network architecture that combines Backbone, DCA-Block, and CWT-Block; Baseline 3 denotes a network architecture that combines Backbone, DCA-Block, and CT-Block; while HMT-Net includes all the modules.

To further elucidate the specific contributions of each module, we analyzed the causes of performance degradation observed in Table 9.

Firstly, the removal of the DCA-Block leads to a noticeable decline in accuracy. This is primarily because the DCA-Block employs dilated convolutions to expand the receptive field, allowing the model to capture global context without increasing computational complexity. In low SNR conditions, local features are often corrupted by noise; the DCA-Block helps mitigate this by aggregating information across broader spatial dimensions, thereby enhancing the model’s robustness against noise interference.

Secondly, the CT-Block plays a pivotal role in modeling the long-range sequential dependencies inherent in convolutional codes. When the CT-Block is removed, the network relies solely on local convolutional features, failing to capture the structural correlations of the code sequences. This deficiency makes it particularly difficult to differentiate between codes with similar generator polynomials but different constraint lengths, resulting in a higher misclassification rate for these difficult categories.

Finally, the CWT-Block is essential for task-specific feature refinement. By leveraging the self-attention mechanism along the channel dimension, the CWT-Block highlights the most discriminative features for each specific task (code rate vs. constraint length). The absence of this module leads to feature redundancy and confusion between tasks, preventing the model from achieving optimal precision in the final classification stage.

## 5. Conclusions

In this paper, we propose a deep learning network, HMT-Net, for the identification of convolutional code parameter based on multi-task learning. HMT-Net consists of a feature sharing module and a task-specific module. The feature sharing module incorporates multi-scale null convolution with multiple attention mechanisms and introduces a Transformer network structure to efficiently capture sequence features. The task-specific module combines channel-level Transformer with convolutional network structures to fully leverage the unique feature representations of each recognition subtask, thereby enhancing classification accuracy. HMT-Net exploits the potential correlation among multiple tasks and achieves complementary information through shared features. This approach not only significantly improves classification accuracy but also reduces computational complexity and resource consumption, effectively enhancing overall performance. Additionally, the study proposes a comprehensive sequence dataset by extracting autocorrelation information from convolutional codes along with WHT transform, which further boosts the performance of convolutional code parameter recognition.

In future work, we will further investigate the recognition of multiple channel codes and utilize self-supervised learning techniques to broaden the applicability of our research. Specifically, we plan to expand the evaluation scope to include a wider variety of convolutional codes, such as systematic convolutional codes, recursive convolutional codes, and cyclic convolutional codes. Furthermore, we will investigate the model’s performance under more complex combinations of generator polynomials and coding parameters to comprehensively validate the generalization ability of HMT-Net across diverse coding structures.

## Figures and Tables

**Table 1:** HMT-Net parameter configurations.

Module	Network Layer	Parameters	Output Dimension
Input: coded signal (dimension: 1×16×16)			
Preproc- essing	Conv	Conv(3 × 3, 64), BN, GELU, MaxPool(2)	64 × 16 × 16
Feature sharing module (in software)	CT-Block 1	Conv(3 × 3, 64) + CWT(64)	64 × 16 × 16
	DCA-Block 1	64-channel convolution with attention mechanism	64 × 16 × 16
	CT-Block 2	Conv(3 × 3, 64) + CWT(64)	64 × 16 × 16
	DCA-Block 2	64-channel convolution with attention mechanism	64 × 16 × 16
Mission- specific module (in software)	CT-Block 3	Conv(3 × 3, 64) + CWT(64)	64 × 16 × 16
	CWT-Block	64-channel, 4-head self- attention mechanism	64 × 16 × 16
	Global average pooling	(1 × 1)	64 × 1
	FC 1	[64 → 256] + GELU activation	256 × 1
	FC 2	[256 → K] + Softmax	K × 1
Output: ith subtask (t∈N) prediction class probability vector (dimension: K×1)			
Dynamic weighting	task weight parameter	Learnable parameters $w\in R^{N}$ EMA update factor α=0.8	N × 1

**Table 2:** Network training hyperparameter configurations.

Parameters	Value
Initial learning rate	0.001
Training cycle	60
Learning Rate Scheduler	ReduceLROnPlateau
Batch size	64
Optimizer	Adam

**Table 3:** The parameters of the convolutional code.

Classification of Tasks	Code Rate	Constraint Length
Convolutional code	$r=\left[1/2,1/3,1/4,2/3,3/4\right]$	$l=\left[3,4,5,6,7,8\right]$

**Table 4:** Comparison of recognition accuracy of HST-Net, HCT-Net, and HMT-Net.

Model	Code Rate Recognition Accuracy	Constraint Length Recognition Accuracy	Average Recognition Accuracy
HST-Net	62.01%	59.21%	60.61%
HCT-Net	64.42%	57.83%	61.13%
**HMT-Net**	**66.31%**	**60.68%**	**63.50%**

Note: The bold text indicates the best performance.

**Table 5:** Per-class accuracy and F1-score analysis (SNR ≥ 0 dB).

Task	Class	Per-Class Accuracy (Recall)	F1-Score
Code Rate	1/2	92.60%	90.52%
	1/3	94.41%	94.03%
	2/3	97.40%	96.48%
	1/4	81.40%	82.06%
	3/4	79.70%	82.04%
	**Macro-Avg**	**89.10%**	**89.03%**
Constraint Length	K = 3	84.02%	84.99%
	K = 4	81.12%	82.35%
	K = 5	81.38%	81.59%
	K = 6	82.68%	82.81%
	K = 7	86.80%	85.35%
	K = 8	84.68%	83.56%
	**Macro-Avg**	**83.45%**	**83.44%**
Note: The bold text indicates the macro-average performance across all classes.			

**Table 6:** Comparison of number of parameters.

Methodologies	Task	Parameter Quantification	Inference Time	FLOPs
HST-Net	Code rate	304.02 K	5.73 ms	18.4 MFLOPs
	Constraint length	304.27 K	5.84 ms	18.4 MFLOPs
HCT-Net	Combined	612.15 K	11.08 ms	36.7 MFLOPs
HMT-Net	Combined	385.16 K	6.94 ms	22.5 MFLOPs

**Table 7:** Multi-network performance comparison.

Model	Code Rate Recognition		Constraint Length Recognition	
	SNR ∈ [0, 20]	SNR ∈ [−20, 20]	SNR ∈ [0, 20]	SNR ∈ [−20, 20]
MAR-Net [26]	93.16%	61.74%	86.21%	56.37%
AMSCN [27]	87.84%	59.98%	84.93%	56.20%
FFMNet [28]	72.38%	51.05%	51.81%	37.58%
ConvLSTM-TFN [29]	86.60%	59.58%	78.15%	53.62%
**HMT-Net**	**95.01%**	**66.31%**	**88.05%**	**60.68%**
	**(1.85%)**	**(4.57%)**	**(1.84%)**	**(4.31%)**

Note: The bold text indicates the proposed method and the best performance.

**Table 8:** Comparison of recognition accuracy between EMA and custom weighting methods.

α	β	Recognition Accuracy	
		Code Rate	Constraint Length
0.1	0.9	30.19%	59.53%
0.2	0.8	59.73%	59.33%
0.3	0.7	64.82%	59.61%
0.4	0.6	**65.77%**	**59.74%**
0.5	0.5	65.39%	58.87%
0.6	0.4	64.83%	57.82%
0.7	0.3	64.97%	53.77%
0.8	0.2	63.89%	42.48%
0.9	0.1	63.21%	24.31%
**EMA**		**66.31%**	**60.68%**

Note: The bold text indicates the optimal results for the fixed weighting scheme and the proposed EMA method.

**Table 9:** HMT-Net ablation experiments.

Module	Backbone	DCA	CT	CWT	Recognition Accuracy	
					Rate	Constraint Length
Baseline 1	✓		✓	✓	63.46%	58.26%
Baseline 2	✓	✓		✓	58.93%	52.29%
Baseline 3	✓	✓	✓		60.08%	55.52%
**HMT-Net**	✓	✓	✓	✓	**66.31%**	**60.68%**
Note: ✓ indicates that the module is included; bold text indicates the best performance.						

## Data Availability

Data are contained within the article.
